# Supplementary material for: Machinability and tribological optimization of origami-inspired Almond Shell–PMMA via RSM, ML, and TOPSIS
Source: PLoS One. 2026 Jan 23;21(1):e0341273. doi: 10.1371/journal.pone.0341273 (PMC12829812; doi:10.1371/journal.pone.0341273)
Supplement: Attachment — Submitted filename: review comments.docx [file pone.0341273.s004.docx]

The revised manuscript titled “**Machinability and Tribological Optimization of Origami-Inspired Almond Shell–PMMA via RSM, ML, and TOPSIS**” has been carefully evaluated along with the detailed responses to reviewer comments. The authors have satisfactorily addressed all major and minor concerns raised during peer review.

The revision demonstrates clear improvements in technical rigor, methodological consistency, and clarity of presentation. Key issues related to experimental design, wear mechanisms, machinability assessment, hybrid optimization workflow, and data support have been properly resolved. The study presents a well-integrated hybrid framework combining RSM, SVM-based machine learning, and TOPSIS for multi-objective optimization, applied to a sustainable almond shell–PMMA composite with origami-inspired architectures.

Based on the satisfactory revisions and the overall quality of the manuscript, it is **recommended for acceptance.**
